# Effects of Parent-Involved Digital Health Interventions on Adiposity-Related Outcomes and Behaviors in Children and Adolescents With Overweight or Obesity: Systematic Review and Meta-Analysis

**DOI:** 10.2196/95757

**Published:** 2026-10-30

**Authors:** Yuan Zheng, Jiafu Huang, Yuhang Peng, Kaifeng Wu, Fenghua Sun, Wendy Huang, Chunxiao Li

**Affiliations:** 1 Physical Education & Sports Science South China Normal University Guangzhou China; 2 School of Physical Education Guangzhou Sport University Guangzhou China; 3 Department of Health and Physical Education The Education University of Hong Kong Hong Kong China (Hong Kong); 4 Department of Sports and Health Sciences Academy of Wellness and Human Development Hong Kong Baptist University Hong Kong China (Hong Kong); 5 Dr. Stephen Hui Research Centre for Physical Recreation and Wellness Hong Kong Baptist University Hong Kong China (Hong Kong); 6 Department of Sport and Exercise Sciences Durham University Durham, County Durham United Kingdom

**Keywords:** parental involvement, technology-based intervention, pediatric weight management, adiposity outcomes, healthy behaviors, mobile phone

## Abstract

**Background:**

Parental involvement in obesity interventions is considered essential for sustainable behavior change in children and adolescents with overweight or obesity; yet, the effectiveness of parent-involved digital health interventions (DHIs) and the influence of different delivery modalities remain unclear.

**Objective:**

This study aimed to synthesize the effects of parent-involved DHIs on adiposity-related outcomes and behaviors and explore potential moderators.

**Methods:**

Nine databases were searched from inception to December 2025 to identify eligible randomized controlled trials (RCTs). Primary outcomes were adiposity-related outcomes such as BMI; secondary outcomes were adiposity-related behaviors, including dietary behavior, physical activity, and sedentary behavior. Standardized mean differences (SMDs; Hedges *g*) on primary outcomes were pooled using random-effects models. Prespecified subgroup analyses examined DHI delivery modalities and parent-involved strategies. Secondary outcomes were synthesized narratively. Risk of bias was assessed using the revised Cochrane Risk of Bias tool for randomized trials (RoB 2), and certainty of evidence was evaluated using GRADE (Grading of Recommendations, Assessment, Development, and Evaluation).

**Results:**

Twenty-nine RCTs (4156 participants) were included, of which 22 contributed to the meta-analysis. Parent-involved DHIs produced a small but statistically significant reduction in BMI-related outcomes compared with controls (SMD=*−*0.11, 95% CI *−*0.19 to *−*0.04; *P*=.004; *I*²=0%). When stratified by control type, the pooled effect remained significant in trials using inactive controls (SMD=*−*0.15, 95% CI *−*0.25 to *−*0.04; *P*=.006; *I*²*=*0%), but not in trials using active controls (SMD=*−*0.07, 95% CI *−*0.19 to 0.04; *P*=.20; *I*²*=*0%). Subgroup differences on DHI delivery modalities and parent-involved strategies were not statistically significant. For adiposity-related behaviors, significant improvements were reported in 5 of 16 trials for dietary behaviors, 2 of 17 for physical activity, and 2 of 12 for sedentary behavior. The risk of bias was generally low to some concerns, and the overall certainty of evidence for BMI-related outcomes was moderate.

**Conclusions:**

Moderate-certainty evidence suggests that parent-involved DHIs may produce a small improvement in BMI-related outcomes among children and adolescents with overweight or obesity. However, evidence for improvements in adiposity-related behaviors remains limited, and neither the optimal digital delivery modality nor the most effective parent-involved strategy has been established. Future research should refine these interventions to identify optimal strategies and confirm their effectiveness.

**Trial Registration:**

PROSPERO CRD420251239829; https://www.crd.york.ac.uk/PROSPERO/view/CRD420251239829

## Introduction

Childhood and adolescent overweight and obesity are chronic, relapsing conditions and constitute one of the most pressing global public health challenges of the 21st century [1]. Beyond excess body weight, pediatric obesity is associated with a wide spectrum of cardiometabolic complications, nonalcoholic fatty liver disease, type 2 diabetes, and adverse mental health outcomes [2]. Recent pooled global estimates indicate that the combined prevalence of overweight and obesity among children and adolescents increased from 8% in 1990 to 20% in 2022, corresponding to more than 390 million affected individuals worldwide [3]. From a life-course perspective, this trend signals a continued expansion of the chronic disease burden in adulthood, as overweight and obesity during childhood and adolescence often persist into adulthood and significantly increase all-cause mortality and the risk of cardiovascular disease [4].

In response to this growing burden, digital health interventions (DHIs) have emerged as a promising strategy for the treatment of pediatric overweight and obesity. Specifically, DHIs for individuals with overweight and obesity typically use digital delivery modalities such as telehealth, mobile apps, and wearable devices to facilitate self-monitoring and behavior change, thereby supporting weight management and improving health outcomes [5,6]. Although some studies report beneficial effects of DHIs on adiposity-related outcomes such as reductions in body weight and BMI, the evidence remains mixed and often limited to modest or short-term improvements in weight status among children and adolescents with overweight or obesity [7,8]. Additionally, accumulating evidence suggests that DHIs alone may be insufficient to achieve sustained behavior change among children and adolescents with overweight or obesity [9].

For children and adolescents, parents serve as the primary caregivers and gatekeepers of health-related behaviors, while children and adolescents’ capacity to independently make and maintain lifestyle-related decisions remains limited [10]. Consistently, interventions that actively engage parents as participants rather than passive supporters demonstrate more consistent improvements in weight-related and behavioral outcomes compared with approaches targeting only children and adolescents [11,12]. Accordingly, researchers have emphasized integrating parents into the design and implementation of DHIs as well as using digital delivery technologies to facilitate their involvement [13]. Indeed, parent-involved strategies in DHIs are diverse, varying in the roles parents assume, the forms of participation, and the activities they undertake within interventions [14]. However, no consensus has been reached regarding which parent-involved DHI strategies are most effective, and further evidence is needed to determine how parents should participate to achieve optimal outcomes in addressing pediatric overweight and obesity.

To date, only a limited number of reviews have examined the effects of parent-involved DHIs on adiposity-related outcomes in children and adolescents with overweight or obesity. To our knowledge, the earliest review addressing parent-involved DHIs was published in 2016 and was limited to parent-focused eHealth interventions [15]. Given the rapid development of digital health technologies over the past decade [16], the relevant evidence needs to be further expanded and updated. More recent reviews have likewise been restricted in scope, focusing on mobile health interventions [17], specific ethnic populations [18], or primarily home-based settings [19]. These limitations in digital health scope, population characteristics, and intervention settings may restrict the generalizability of current evidence. Moreover, although these reviews have highlighted the potential benefits of involving parents in DHIs, they have not fully clarified whether variations in parental involvement characteristics, such as intervention target, mode of engagement, and participation setting, are associated with differences in intervention outcomes. Digital delivery modality may represent a potential source of heterogeneity because different modalities support different intervention functions [20]. For example, telehealth may facilitate professional counseling and intervention monitoring, whereas exergaming or active video gaming may primarily promote physical activity engagement [8]. However, whether specific digital delivery modalities within parent-involved DHIs are associated with differences in intervention effects remains unclear in existing reviews.

Therefore, this systematic review with meta-analysis aimed to provide a comprehensive evaluation of parent-involved DHIs for treating overweight and obesity in children and adolescents. Specifically, we examined the pooled effect of parent-involved DHIs on adiposity-related outcomes (eg, BMI or BMI *z* score) and examined whether DHI delivery modalities and parent-involved strategies moderate intervention effects. Dietary intake, physical activity, and sedentary behavior were synthesized narratively as complementary outcomes to provide further insight into potential behavioral pathways underlying intervention effects. This review provides an updated estimate of the effectiveness of parent-involved DHIs and an exploratory assessment of intervention characteristics that may be associated with variation in outcomes.

## Methods

### Protocol Registration and Reporting Guideline

This review follows the PRISMA (Preferred Reporting Items for Systematic Reviews and Meta-Analyses) 2020 guideline [21]. The PRISMA checklist is provided in Multimedia Appendix 1. The protocol was registered prospectively in PROSPERO (International Prospective Register of Systematic Reviews; registration ID: CRD420251239829).

### Information Sources and Search Strategy

A comprehensive literature search was conducted on December 4, 2025, across the following electronic databases: MEDLINE (via Ovid), Embase, Emcare (via Ovid), CINAHL (via EBSCOhost), PsycInfo (via EBSCOhost), ProQuest Central, Web of Science (Core Collection), Scopus, and SPORTDiscus (via EBSCOhost). The search combined controlled vocabulary (eg, MeSH terms) and free-text keywords for the population, intervention, and study design, using Boolean operators (AND/OR) and adapting to each database’s indexing. Full search strategies for all databases are provided in Multimedia Appendix 2. No limits were placed on publication date or status, and only English-language publications were included.

### Eligibility Criteria

Inclusion criteria were defined a priori according to the PICOS (Population, Intervention, Comparison, Outcomes, and Study Design) framework. Studies were included if they met all of the following criteria.

Regarding population, children and adolescents ≤18 years of age with overweight or obesity, as defined by international or national growth references (eg, World Health Organization or International Obesity Task Force), and/or their parents.

Regarding intervention, parent-involved DHIs. Specifically, interventions met the following criteria: (1) digital technology was the main means of delivery for the intervention, including wearable devices, mobile phone apps, text messaging services, websites, active video gaming, and/or telehealth [6,8]; and (2) parental involvement, as operationalized in parent-involved DHIs, was defined as parents serving as participants in the intervention [22]. Eligible interventions required parents to engage in one or more intervention-related activities, such as receiving intervention content, participating in counseling or coaching sessions, completing assigned activities, monitoring children’s and adolescents’ behaviors, or supporting intervention implementation. Either or both parents could participate.

Regarding control, usual care, waitlist control, or any alternative intervention not incorporating both a digital delivery component and parental involvement.

Regarding outcome, primary outcomes were changes in adiposity-related outcomes, prioritizing BMI *z* score, followed by BMI, and other BMI-related measures when necessary [23]. Secondary outcomes included changes in dietary intake, physical activity, and sedentary behavior that are closely tied to adiposity [1,19].

Regarding study design, individual randomized or cluster randomized controlled trials (RCTs).

The exclusion criteria included the following: (1) interventions targeting children or adolescents only, without an active parental involvement component; (2) interventions in which parents only received general information, were notified about the intervention, or completed assessments without participating in intervention activities; (3) interventions without a primary digital delivery component; and (4) nonrandomized studies, protocols, reviews, conference abstracts, and studies without relevant adiposity-related or behavioral outcomes.

### Study Selection

All database search results were imported into Zotero software (version 8.0.2, 64-bit; Corporation for Digital Scholarship) for deduplication. Three reviewers (YZ, YP, and KW) independently screened titles and abstracts according to the predefined eligibility criteria, and clearly irrelevant records were excluded at this stage. Full texts of potentially eligible studies were independently assessed by the same reviewers. Any disagreements were resolved through discussion, with adjudication by a fourth reviewer (JH) when necessary.

### Data Extraction

Two reviewers (YZ and KW) independently extracted data from each included study using a standardized form: study characteristics (author, year, and country), participant characteristics (sample size and age range), study design (ie, individual vs cluster RCT), intervention details (eg, duration, theoretical framework, and group), digital delivery modalities (eg, mobile phone apps, text messaging, and websites), parent-involved strategies (eg, where involvement occurs and how parents engage with intervention content), and primary and secondary outcomes measured. For digital delivery modalities and parent-involved strategies, 2 reviewers (YZ and KW) independently classified digital delivery modalities and parent-involved strategies according to the predefined operational criteria (Multimedia Appendix 3 [6,14-16,24-32]). All extracted data were cross-checked for accuracy, and any discrepancies between reviewers were resolved by referring back to the original studies or by involving a third reviewer (JH).

### Risk of Bias Assessment

Risk of bias was assessed using the revised Cochrane Risk of Bias tool for randomized trials (RoB 2) [33]. Individually randomized parallel group trials were evaluated using the RoB 2 tool for individually randomized trials [34], while cluster RCTs were assessed using the RoB 2 tool for cluster randomized trials [35]. The RoB 2 tool evaluates 5 domains; each domain was judged as “low risk of bias,” “some concerns,” or “high risk of bias.” Two reviewers (YZ and KW) independently conducted the assessments using the official RoB 2 Excel tool. Disagreements were resolved through discussion, with adjudication by a third reviewer (JH) when necessary.

### Statistical Analysis

Meta-analyses were carried out using Review Manager (RevMan, version 5.4.1; The Cochrane Collaboration). All analyses were conducted at the study level, with each trial contributing a single effect size per outcome. For adiposity-related outcomes, means, SDs, and the sample sizes for the intervention and control groups were extracted at baseline, postintervention, and follow-up. When multiple follow-up time points were reported, outcomes closest to the end of the intervention were selected. Change-from-baseline means and SDs were calculated from reported baseline and postintervention values, assuming a correlation coefficient of 0.5 [33]. When means or SDs were not directly reported, they were calculated or estimated from available summary statistics, including medians, sample sizes, SEs, 95% CIs, or IQRs, where applicable. All calculations and data transformations were independently verified by 2 reviewers (YZ and KW). Because BMI-related outcomes were reported using different metrics across studies, intervention effects were summarized using standardized mean differences (SMDs; Hedges *g*) with 95% CIs [36]. All meta-analyses were performed using random-effects models to account for anticipated clinical and methodological heterogeneity across studies [37]. Statistical heterogeneity was assessed using the *I*² statistic, with values of <25%, 25%-50%, and >50% indicating low, moderate, and high heterogeneity, respectively [38].

Following the Cochrane Handbook, systematic reviews should clearly distinguish between active and inactive comparator conditions, as they represent different comparison contexts that may influence both the magnitude and interpretation of intervention effect estimates [33]. Previous methodological research has further cautioned that combining different comparator conditions may result in ambiguous or misleading estimates [39]. Therefore, we conducted a supplementary subgroup analysis by control type to contextualize the pooled effects under different comparator conditions.

Prespecified subgroup analyses were conducted to examine potential effect modification by DHI delivery modalities [8] and parent-involved strategies, when at least two studies were available per category [33]. Digital delivery modalities were classified into mobile phone apps, text messaging, websites, exergaming or active video gaming, telehealth, telephone, or hybrid combination interventions. The classification was based on the primary digital channel and intervention function described in the original studies. Because some modalities overlap conceptually, classification was not based on the device alone; instead, studies were assigned according to the modality that best represented the primary delivery model [6,8]. The included RCTs generally reported clear primary delivery modalities; for example, interventions were classified as telephone when coaching or counseling was primarily delivered through telephone calls [40], and as text messaging when intervention content was mainly delivered through text messages [41]. Studies incorporating two or more equally important primary delivery modalities were classified as hybrid combination interventions [20]. Using a multidimensional framework, parent-involved strategies were classified by locus (setting of involvement), style (degree of formality), action (mode of engagement), and actor (intervention target) [14]. The categories within each dimension were operationally defined based on the characteristics of included parent-involved DHIs. The style dimension includes formal and informal parental involvement; however, style was not examined as an effect modifier because eligibility criteria required structured parental involvement. Therefore, all included interventions were classified as formal involvement. Operational definitions and classification criteria for DHI delivery modalities and parent-involved strategies are provided in Multimedia Appendix 3.

### Publication Bias Assessment

When at least 10 studies were available for a given meta-analysis [42], potential publication bias and small-study effects were assessed by visually inspecting a funnel plot of effect size against SE.

### Certainty of Evidence

The certainty of evidence for primary outcomes was assessed using the GRADE (Grading of Recommendations, Assessment, Development, and Evaluation) approach at the outcome level [33]. Evidence derived from RCTs was initially rated as high certainty and downgraded based on 5 domains: risk of bias, inconsistency, indirectness, imprecision, and publication bias. Final certainty ratings were classified as high, moderate, low, or very low. GRADE assessments were independently performed by 2 reviewers (YZ and KW), with disagreements resolved through discussion or adjudication by a third reviewer (JH).

## Results

### Study Selection

The database searches identified 3713 records. After removal of duplicates, 1940 unique records were retained for title and abstract screening, of which 1820 were excluded as clearly irrelevant. Subsequently, 120 full-text papers were retrieved and assessed for eligibility. Of these, 91 papers were excluded for various reasons. Ultimately, 29 RCTs met the inclusion criteria and were included in the qualitative synthesis, of which 22 trials had sufficient data for inclusion in the meta-analyses. The study selection process is illustrated in Figure 1, and detailed reasons for full-text exclusion and inclusion are presented in Multimedia Appendix 4 [3,40,41,43-68].

**Figure 1:**
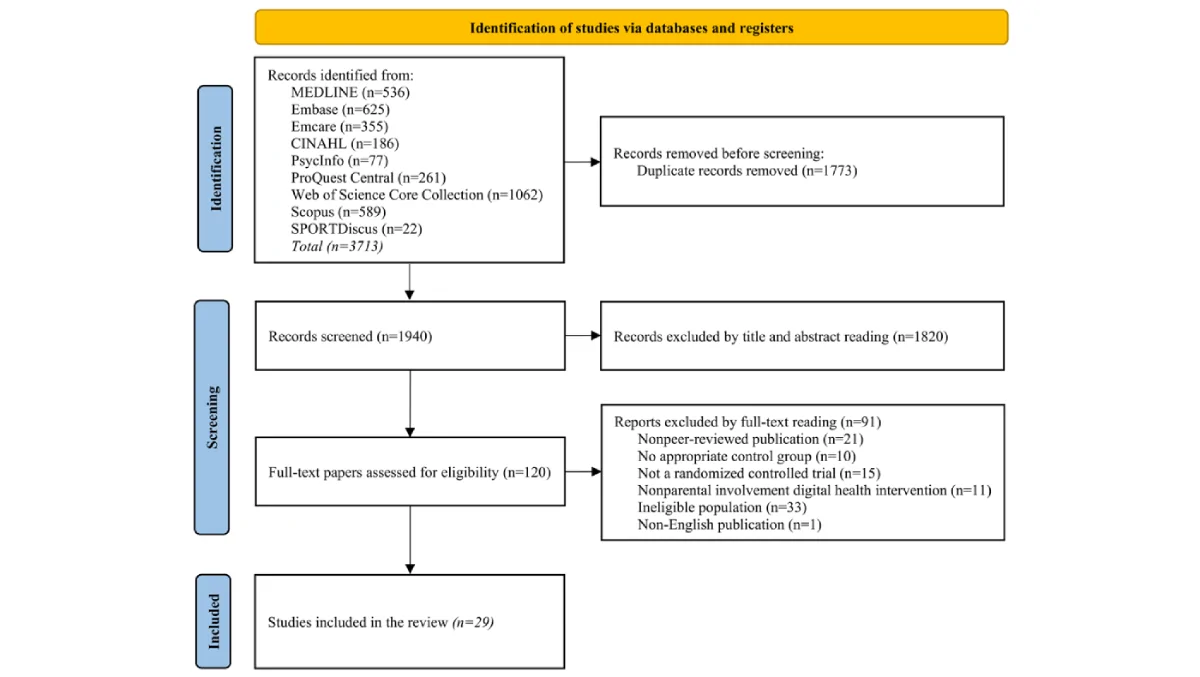
PRISMA (Preferred Reporting Items for Systematic Reviews and Meta-Analyses) flowchart of study selection.

### Study Characteristics of Included Studies

The 29 included trials were published between 2004 and 2025 and were conducted in 11 different countries. Combined, these studies enrolled 4156 children and adolescents with overweight or obesity; individual sample sizes ranged from 20 to 1120 participants, with a mean sample size of 143.3 (SD 220.6) participants per study. Participant ages spanned from 3 to 18 years. Of the 29 studies, 3 trials had multiple intervention arms [43-45]. Intervention durations varied widely, from 5 weeks to 24 months. Fourteen studies reported using an explicit theoretical framework to guide the intervention (most often Social Cognitive Theory or behavior change techniques) [40,43-55].

Regarding the DHI delivery modalities, 6 trials used websites [44,45,47,52,56,57], 4 trials delivered interventions via a mobile phone app [46,58-60], 4 trials used telephone [40,49,50,55], 3 trials primarily used text messaging [41,61,62], 3 trials used telehealth [3,48,51], 1 trial evaluated an active video gaming approach [63], and 8 trials implemented a hybrid approach that combined two or more delivery modalities [43,53,54,64-68].

Using the predefined classification framework for parental involvement (see the Methods section), we characterized each intervention across 4 key dimensions. With respect to the locus of parent involvement, parental participation occurred exclusively in the home setting in 4 trials [52,57,62,67], and parental participation occurred across outpatient and home settings in 17 trials [3,40,41,43,47-51,55,56,58-61,65,66]. In the remaining 8 trials, parental participation occurred across school or community, and home settings [44-46,53,54,63,64,68]. In terms of involvement style, all 29 trials adopted a formal structure for parent participation. Regarding the mode of action, 20 trials involved synchronous or quasi-synchronous interaction between parents and intervention personnel [3,40,41,43,45-51,53-55,60,61,65-68], 8 trials emphasized self-directed parent engagement without real-time professional interaction [44,52,56-59,63,64], and only 1 trial adopted a fully indirect, noninteractive delivery model [62]. Finally, concerning the primary actor targeted by the intervention, 11 trials were parent-focused [3,40,41,44,46,50,56,60-62,65], 14 trials were parent-led joint interventions [43,45,48,49,51,52,54,55,57-59,66-68], and 4 trials were child- and adolescent-targeted interventions with parent support that included parents in a supportive capacity [47,53,63,64].

Following the Cochrane Handbook, control-group designs can be broadly categorized as active control (eg, alternative intervention variants or different therapeutic approaches) or inactive (eg, waitlist control, no treatment, or usual or standard care) [33,39]. Among the 29 included trials, 14 trials used active controls [3,45,47-51,53,57,58,62-64,68], whereas 15 trials used inactive controls, including 7 waitlist controls [43,46,52,55,59,66,67], 4 no-intervention controls [44,54,56,61], and 4 usual or standard care controls [40,41,60,65].

All 29 studies assessed at least one BMI-related outcome (BMI, BMI *z* score, or BMI percentile), and 10 studies also reported central adiposity measures, including waist circumference (WC) and waist height ratio (WHR) [43,45,46,51-54,58,64,67]. Most trials collected secondary outcomes: 16 assessed dietary behaviors [3,41,43,45,48-53,55,58,59,61,67,68], 17 measured some form of physical activity [3,41,43,45,48-52,56,58,59,62-64,67,68], and 12 evaluated sedentary behavior (typically screen time) [3,41,45,49,50,52,53,55,56,59,67,68]. The main characteristics of the included studies are summarized in Table 1.

**Table 1:** Study characteristics.

Author, year, country	Children and adolescents		Study design	Intervention details			Digital delivery modalities		Parent-involved strategies					Outcome measures	
	N	Age (y)		Duration	Theoretical framework	Group		Locus		Style	Action	Actor	Primary		Secondary
Ahmad et al [46], 2018, Malaysia	134	8-11	IRCT^a^	6 mo	SCT^b^	IG^c^: REDUCE^d^ program; CG^e^: waitlist control	Mobile phone apps	School/community + home		Formal	Syn/quasi-syn^f^ interaction	Parent-focused	BMI *z* score, WC^g^		N/A^h^
Armstrong et al [41], 2018, United States	101	5-12	IRCT	3 mo	N/A	IG: text messages; CG: standard care	Text messages	Outpatient + home		Formal	Syn/quasi-syn interaction	Parent-focused	BMI *z* score		PA^i^, SB^j^
Baños et al [47], 2019, Spain	47	8-12	IRCT	10 wk	CBT^k^	IG: CBT-E^l^ group; CG: CBT group	Websites	Outpatient + home		Formal	Syn/quasi-syn interaction	Child- and adolescent-targeted + parent support	BMI *z* score		N/A
Bohlin et al [40], 2017, Sweden	37	5-14	IRCT	18 mo	CBT	IG: telephone coaching; CG: usual care	Telephone	Outpatient + home		Formal	Syn/quasi-syn interaction	Parent-focused	BMI *z* score		N/A
Chai et al [43], 2021, Australia	31	4-11	IRCT	3 mo	BCT^m^	IG: telehealth group; CG: waitlist control	Telehealth, websites, mobile phone apps	Outpatient + home		Formal	Syn/quasi-syn interaction	Parent-led joint	BMI *z* score, WC		DB^n^, PA
Damayanti et al [64], 2025, Indonesia	45	9-13	CRCT^o^	2 mo	N/A	IG: online nutrition, PA, + parents; CG: online nutrition, PA	Websites, mobile phone apps	School/community + home		Formal	Self-directed completion	Child- and adolescent-targeted + parent support	BMI *z* score, WC		PA
Davis et al [48], 2013, United States	42	6-11	IRCT	8 mo	BCT	IG: telemedicine; CG: physician visit	Telehealth	Outpatient + home		Formal	Syn/quasi-syn interaction	Parent-led joint	BMI *z* score		DB, PA
Davis et al [49], 2024, United States	135	5-14	CRCT	8 mo	SCT, CBT	IG: iAmHealthy^p^; CG: newsletter	Telephone	Outpatient + home		Formal	Syn/quasi-syn interaction	Parent-led joint	BMI *z* score		DB, PA, SB
Estabrooks et al [50], 2009, United States	99	8-12	IRCT	12 mo	SET^q^	IG: FC^r^-group + FC-IVR^s^; CG: FC-workbook	Telephone	Outpatient + home		Formal	Syn/quasi-syn interaction	Parent-focused	BMI *z* score		PA, SB, DB
Fiechtner et al [61], 2016, United States	489	6-12	CRCT	12 mo	N/A	IG: CDS^t^ + a family self-guided behavior; CG: no intervention	Text messages	Outpatient + home		Formal	Syn/quasi-syn interaction	Parent-focused	BMI *z* score		DB
Fleischman et al [51], 2016, United States	36	10-17	IRCT	6 mo	CBT	IG: PCP^u^ visits + specialist tele-visits; CG: PCP visits only	Telehealth	Outpatient + home		Formal	Syn/quasi-syn interaction	Parent-led joint	BMI *z* score, WC		Diet, PA
Johansson et al, 2020 [60], Sweden	28	5-12	IRCT	6 mo	N/A	IG: mHealth^v^ + standard care; CG: standard care	Mobile phone apps	Outpatient + home		Formal	Syn/quasi-syn interaction	Parent-focused	BMI *z* score		N/A
Jones et al [44], 2023, United Kingdom	224	4-5; 10-11	CRCT	12 mo	SCT	IG: web-based intervention; CG: no intervention	Websites	School/community + home		Formal	Self-directed completion	Parent-focused	BMI *z* score		N/A
Kim et al [62], 2016, South Korea	42	7-12	IRCT	5 wk	N/A	IG: Y Health Coaching + parents; CG: Y Health Coaching only	Text messages	Home		Formal	Fully indirect, noninteractive	Parent-focused	BMI		PA
Lee et al [54], 2017, Hong Kong, China	115	8-16	CRCT	6 mo	SLT^ac^	IG: structured SBWMP^ad^; CG: no intervention	Mobile phone apps, telephone, text messages	School/community + home		Formal	Syn/quasi-syn interaction	Parent-led joint	BMI, WHR^ae^		N/A
Maddison et al [52], 2014, New Zealand	230	9-12	IRCT	6 mo	BET^w^	IG: SWITCH^x^ intervention package; CG: waitlist control	Websites	Home		Formal	Self-directed completion	Parent-led joint	BMI *z* score, WC		PA, SB, DB
Nguyen et al [53], 2012, Australia	107	13-16	IRCT	12 mo	CBT	IG: Loozit^y^ + ATC^z^; CG: Loozit only	Telephone, text messages	School/community + home		Formal	Syn/quasi-syn interaction	Child- and adolescent-targeted + parent support	BMI *z* score, WC		SB, DB
Pbert et al [3], 2024, United States	462	8-12	CRCT	12 mo	N/A	IG: Fitline coaching; CG: Fitline workbook	Telehealth	Outpatient + home		Formal	Syn/quasi-syn interaction	Parent-focused	BMI *z* score		PA, SB, DB
Ptomey et al [68], 2015, United States	20	11-18	IRCT	2 mo	N/A	IG: eSLD^aa^ delivered + portion-controlled meals; CG: conventional diet	Wearable devices, mobile phone apps	School/community + home		Formal	Syn/quasi-syn interaction	Parent-led joint	BMI		PA, SB, EI^ab^
Resnicow et al [65], 2024, United States	1120	3-11	CRCT	24 mo	N/A	IG: multicomponent MI^af^; CG: usual care	Telehealth, websites, telephone, text messages	Outpatient + home		Formal	Syn/quasi-syn interaction	Parent-focused	BMI %p95^ag^		N/A
Thorén et al [66], 2024, Sweden	65	5-12	IRCT	6 mo	N/A	IG: Web-COP^ah^ intervention; CG: waitlist control	Wearable devices, websites, telephone	Outpatient + home		Formal	Syn/quasi-syn interaction	Parent-led joint	BMI *z* score		N/A
Trost et al [63], 2014, United States	69	8-12	CRCT	4 mo	N/A	IG: program^ai^ + active video games; CG: program^ai^ only	Active video gaming	School/community + home		Formal	Self-directed completion	Child- and adolescent-targeted + parent support	BMI *z* score		PA
Tugault-Lafleur et al [59], 2023, Canada	156	10-17	IRCT	3 mo	N/A	IG: Aim2Be^aj^ app with a live coach; CG: waitlist control	Mobile phone apps	Outpatient + home		Formal	Self-directed completion	Parent-led joint	BMI *z* score		PA, DB, SB
Umano et al [58], 2024, Italy	29	8-11	IRCT	6 mo	N/A	IG: standard treatment + smartphone app; CG: standard treatment only	Mobile phone apps	Outpatient + home		Formal	Self-directed completion	Parent-led joint	BMI *z* score, WHR		DB, PA
Varagiannis et al [45], 2021, Greece	61	8-12	IRCT	6 mo	SCT	IG: web-based (Group 3); CG: group-based sessions (Group 1)	Websites	School/community + home		Formal	Syn/quasi-syn interaction	Parent-led joint	BMI *z* score, WC *z* score		DB, PA, SB
Wald et al [56], 2018, United States	30	3-7	IRCT	12 mo	N/A	IG: group sessions + website access; CG: no intervention	Websites	Outpatient + home		Formal	Self-directed completion	Parent-focused	BMI *z* score		Nutrition, PA
White et al [57], 2004, United States	57	11-15	IRCT	6 mo	N/A	IG: behavioral group; CG: control condition	Websites	Home		Formal	Self-directed completion	Parent-led joint	BMI		N/A
Wright et al [55], 2013, United States	43	9-12	IRCT	3 mo	SCT	IG: IVR-based automated program; CG: waitlist control	Telephone	Outpatient + home		Formal	Syn/quasi-syn interaction	Parent-led joint	BMI *z* score		DB, SB
Zhu et al [67], 2025, Australia	102	7-13	IRCT	10 wk	N/A	IG: web-based program + phone coaching; CG: waitlist control	Websites, telephone	Home		Formal	Syn/quasi-syn interaction	Parent-led joint	BMI *z* score, WC		DB, PA, SB

^a^IRCT: individually randomized controlled trial.

^b^SCT: social cognitive theory.

^c^IG: intervention group.

^d^REDUCE: reorganize diet, unnecessary screen time, and exercise.

^e^CG: control group.

^f^Syn/quasi-syn: synchronous/quasi-synchronous.

^g^WC: waist circumference.

^h^N/A: not applicable.

^i^PA: physical activity.

^j^SB: sedentary behavior.

^k^CBT: cognitive-behavioral theory.

^l^CBT-E: cognitive and behavioral treatment supported by the electronic intelligent therapy obesity web platform.

^m^BCT: behavior change theory.

^n^DB: dietary behavior.

^o^CRCT: cluster randomized controlled trial.

^p^iAmHealthy: a family-based obesity intervention delivered fully via videoconferencing consisting of group and individual sessions.

^q^SET: social-ecological theory.

^r^FC: family connections.

^s^IVR: interactive voice response.

^t^CDS: clinician decision support.

^u^PCP: primary care provider.

^v^mHealth: mobile health.

^w^BET: behavioral economics theory.

^x^SWITCH: screen-time weight-loss intervention targeting children at home.

^y^Loozit: a low-intensity, behavioral family lifestyle intervention.

^z^ATC: additional therapeutic contact.

^aa^eSLD: enhanced Stop Light Diet.

^ab^EI: energy intake.

^ac^SLT: social learning theory.

^ad^SBWMP: school-based weight management program.

^ae^WHR: waist height ratio.

^af^MI: motivational interviewing.

^ag^BMI%p95: BMI as a percent of the 95th percentile.

^ah^Web-COP: an intervention including group sessions and a web-based treatment.

^ai^Program: a comprehensive family-based pediatric weight management program.

^aj^Aim2Be: a gamified lifestyle app designed to promote lifestyle behavior changes.

### Risk of Bias

The results of the risk of bias assessment are presented in Figure 2 (panel A for individual RCTs and panel B for cluster RCTs), and the judgment rationales are provided in Multimedia Appendix 5. Among the 21 individual RCTs, 11 were rated as low risk of bias, and 10 as having some concerns. Among the 8 cluster RCTs, 4 were rated as low risk, 2 as having some concerns, and 2 as high risk. The most common weaknesses were incomplete reporting of allocation concealment and the potential for recruitment or baseline imbalances in cluster trials, and the lack of blinding coupled with suboptimal handling of deviations from the intervention protocol. Ten studies also had possible detection bias, since outcome assessors were aware of participants’ group assignments in some trials.

**Figure 2:**
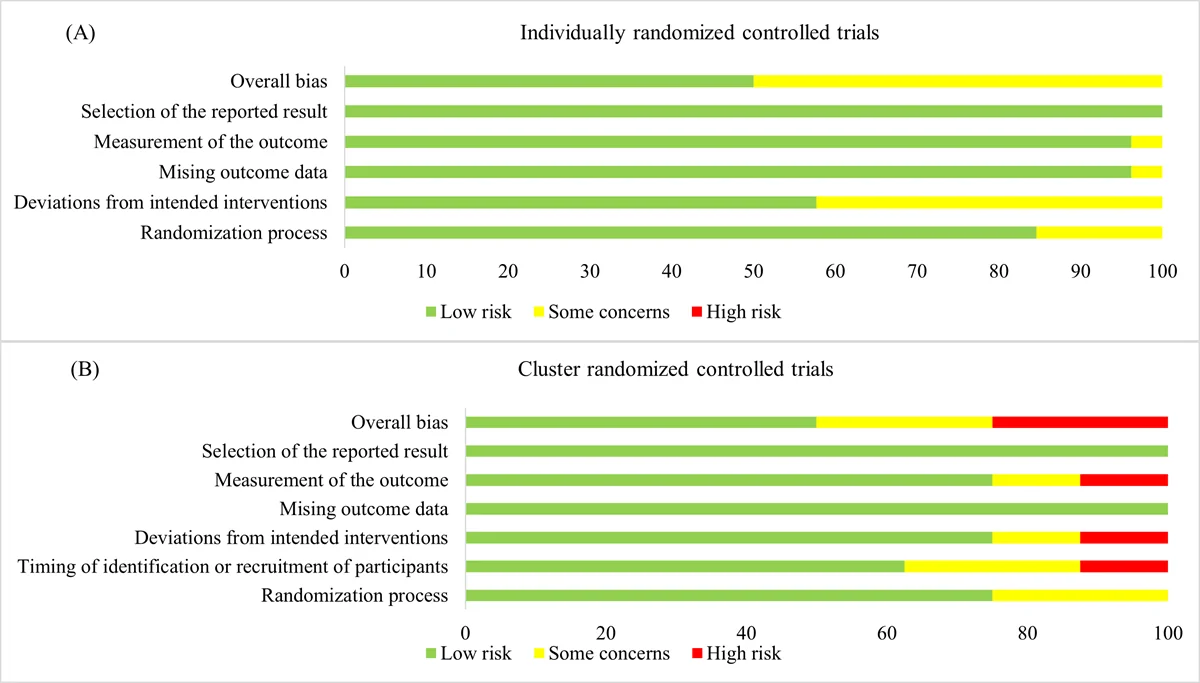
Risk of bias summary.

### Synthesis of the Primary Outcome

#### Descriptive Synthesis

Seven of the 29 RCTs were not included in the quantitative meta-analysis because baseline or postintervention means and SD for BMI-related outcomes were unavailable. Findings across the 7 trials were mixed. Three trials reported significantly greater reductions in BMI or BMI *z* score in the parent-involved DHI groups compared with controls [57,60,66]. One trial showed a trend toward greater BMI reduction with the intervention, although the between-group difference was not statistically significant [54]. The remaining 3 trials reported no significant differences between groups in changes in BMI, BMI *z* score, or BMI%p95 (BMI as a percent of the 95th percentile) [40,41,65].

#### Meta Analysis

A total of 22 RCTs (1379 participants in intervention groups; 1254 in control groups) contributed data to the meta-analysis for BMI-related outcomes. In accordance with the NICE (National Institute for Health and Care Excellence) guideline NG246 [23], BMI *z* score was prioritized for quantitative synthesis, with BMI used when BMI *z* score was not available. Of the 22 included trials, 20 provided BMI *z* scores. As shown in Figure 3 [3,43-53,55,56,58,59,61-64,67,68], parent-involved DHIs yielded a small but statistically significant reduction in BMI-related outcomes compared to control conditions (SMD=*−*0.11, 95% CI *−*0.19 to *−*0.04; Z=2.90, *P*=.004), with no observed heterogeneity (*χ*²_21_=8.64, *P*=.99; *I*²=0%). A sensitivity analysis restricted to trials reporting BMI *z* scores yielded a similar pooled effect (SMD=*−*0.12, 95% CI *−*0.19 to *−*0.04; Z=2.88, *P*=.004), with no evidence of heterogeneity (*χ*²_19_=8.62, *P*=.98; *I*²=0%; Figure S1 in Multimedia Appendix 6 [3,43-53,55,56,58,59,61-64,67]). Considering the rapid evolution of digital health technologies, an additional sensitivity analysis was conducted by restricting the analysis to RCTs published between 2016 and 2025 to assess whether the primary finding remained consistent among more recently published trials. The pooled effect remained statistically significant and was comparable to the primary analysis (SMD=*−*0.13, 95% CI *−*0.22 to *−*0.05; *P*=.003), with no evidence of heterogeneity (*χ*²_15_=6.25, *P*=.98; *I*²=0%), indicating that the observed effect was consistent in more recent DHIs (Figure S2 in Multimedia Appendix 6).

Additional analyses were conducted by stratifying trials according to control type to clarify the relative intervention effects. In trials with active controls (k=13), the pooled effect was not statistically significant (SMD=*−*0.07, 95% CI *−*0.19 to 0.04; Z=1.28, *P*=.20), with no evidence of heterogeneity (*χ*²_12_=4.19, *P*=.98; *I*²*=*0%). In contrast, trials with inactive controls (k=9) demonstrated a significant pooled effect (SMD=*−*0.15, 95% CI *−*0.25 to *−*0.04; Z=2.76, *P*=.006), with no evidence of heterogeneity (*χ*²_8_=3.58, *P*=.89; *I*²=0%). No statistically significant differences were observed between control types (*P*=.35).

**Figure 3:**
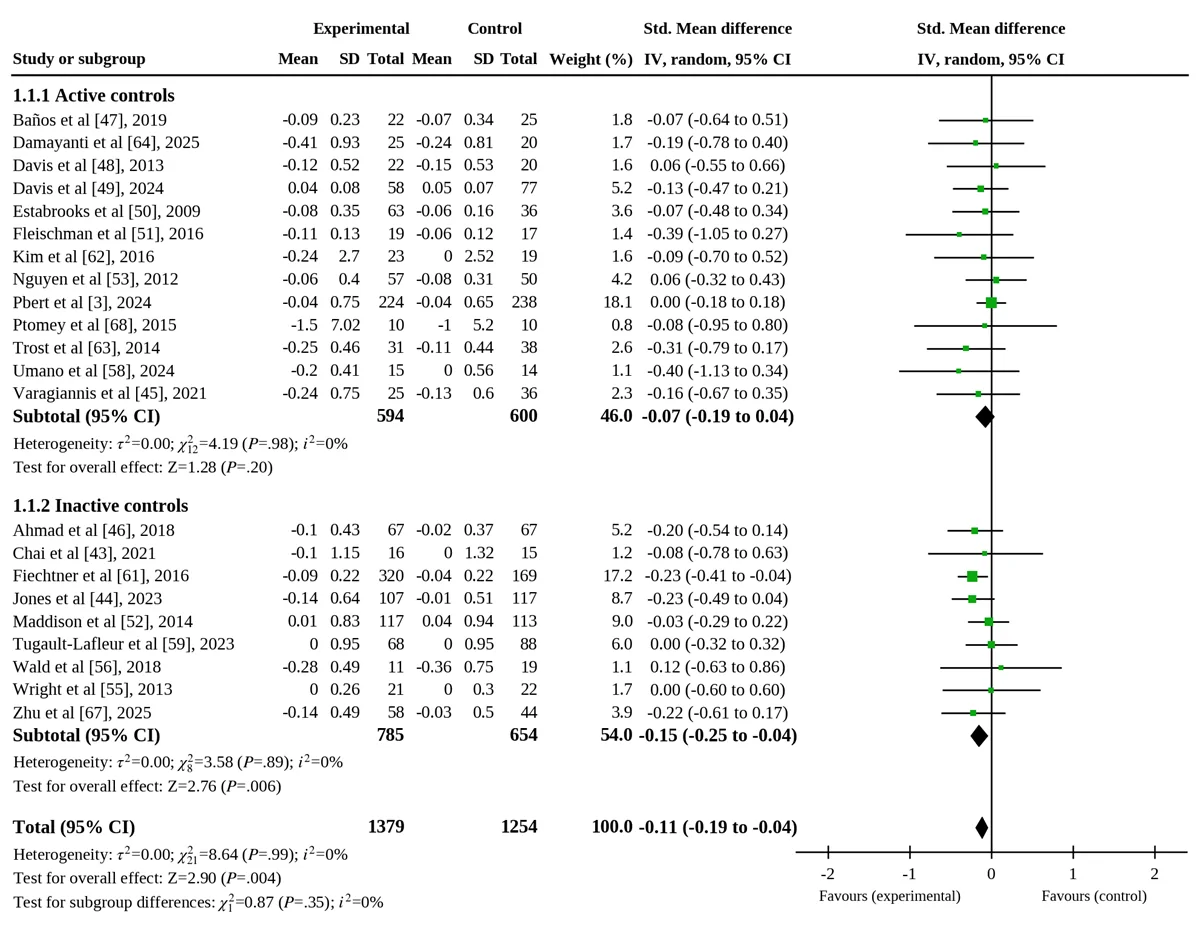
Forest plot of the effects of parent-involved DHIs on BMI-related outcomes [3,43-53,55,56,58,59,61-64,67,68]. Std: standard; DHI: digital health intervention.

#### Subgroup Analysis

The subgroup analysis results based on DHI delivery modalities are presented in Table 2. Among the 22 RCTs included in the meta-analysis, 1 study using active video gaming was excluded from subgroup comparisons because it was the only study in that category [63]. The remaining 21 RCTs were categorized into 6 modalities. Differences in effect sizes across the 6 modalities were not statistically significant (*χ*²_5_=2.45, *P*=.78). While the subgroup of interventions delivered via text messaging demonstrated a significant pooled effect (k=2; SMD=*−*0.22, 95% CI *−*0.39 to *−*0.04; *P*=.02), no significant effects were observed for other subgroups.

The subgroup analysis results based on parent-involved strategies are presented in Table 3. Subgroup analyses were conducted according to the locus of parental involvement. Studies were classified as home-based (k=3), outpatient and home-based (k=12), and school or community and home-based (k=7). There were no significant differences between these 3 subgroups (*χ*²_2_=0.68, *P*=.71). When categorized by action, synchronous or quasi-synchronous interaction (k=14) produced a small yet statistically significant effect (SMD=*−*0.11, 95% CI *−*0.21 to *−*0.02; *P*=.02). Subgroup differences were not significant (*χ*²_1_=0.02, *P=*.90). By parental involvement actor, parent-focused DHIs showed a significant effect (k=7; SMD=−0.13, 95% CI −0.23 to −0.02; *P*=.02), whereas parent-led joint and child- and adolescent-targeted interventions with parent support did not. There was no significant subgroup difference among these 3 categories (*χ*²_2_=0.17, *P=*.92).

**Table 2:** Subgroup analysis for DHI^a^ delivery modalities. Total number of studies included in subgroup comparisons (k=21).

DHI delivery modalities	k^b^ (nI^c^/nC^d^)	SMD^e,f^ (95% CI)	*I*²^g^ (%)	*P* value
Mobile phone apps	3 (150/169)	−0.12 (*−*0.34 to 0.10)	0	.29
Websites	5 (282/310)	−0.11 (*−*0.28 to 0.05)	0	.16
Telehealth	3 (265/275)	−0.02 (*−*0.19 to 0.15)	0	.81
Telephone	3 (142/135)	−0.09 (*−*0.33 to 0.15)	0	.47
Text messaging	2 (343/188)	−0.22 (*−*0.39 to *−*0.04)	0	.02
Hybrid combination	5 (166/139)	−0.09 (*−*0.32 to 0.13)	0	.41

^a^DHI: digital health intervention.

^b^k: number of studies.

^c^nI: number of participants in the intervention group.

^d^nC: number of participants in the control group.

^e^SMD: standardized mean difference.

^f^Hedges *g*.

^g^*I*²: heterogeneity statistic.

**Table 3:** Subgroup analysis for parent-involved strategies^a^.

Parent-involved strategies			k^b^ (nI^c^/nC^d^)		SMD^e,f^ (95% CI)		*I*²^g^ (%)		*P* value
Locus									
	Home-based	3 (198/176)		−0.09 (−0.29 to 0.11)		0		.38	
	Outpatient and home-based	12 (859/740)		−0.10 (−0.20 to 0.00)		0		.06	
	School or community and home-based	7 (322/338)		−0.17 (−0.32 to −0.02)		0		.03	
Action									
	Self-directed completion	7 (374/409)		−0.12 (−0.26 to 0.02)		0		.09	
	Synchronous/quasi-synchronous interaction	14 (982/826)		−0.11 (−0.21 to −0.02)		0		.02	
Actor									
	Parent-focused	7 (815/665)		−0.13 (−0.23 to −0.02)		0		.02	
	Parent-led joint	11 (429/456)		−0.10 (−0.23 to 0.04)		0		.16	
	Child- and adolescent-targeted interventions with parent support	4 (135/133)		−0.10 (−0.34 to 0.14)		0		.42	

^a^Total number of studies included in subgroup comparisons (k=22). For the action dimension, 1 study classified as fully indirect or noninteractive was excluded from subgroup comparison because it was the only study in that category.

^b^k: number of studies.

^c^nI: number of participants in the intervention group.

^d^nC: number of participants in the control group.

^e^SMD: standardized mean difference.

^f^Hedges *g*.

^g^*I*²: heterogeneity statistic.

#### Publication Bias

Visual inspection of the funnel plot did not indicate substantial asymmetry (Figure 4), suggesting no evidence of publication bias for the primary outcome. Therefore, the pooled effect estimates were unlikely to be substantially influenced by small-study effects.

**Figure 4:**
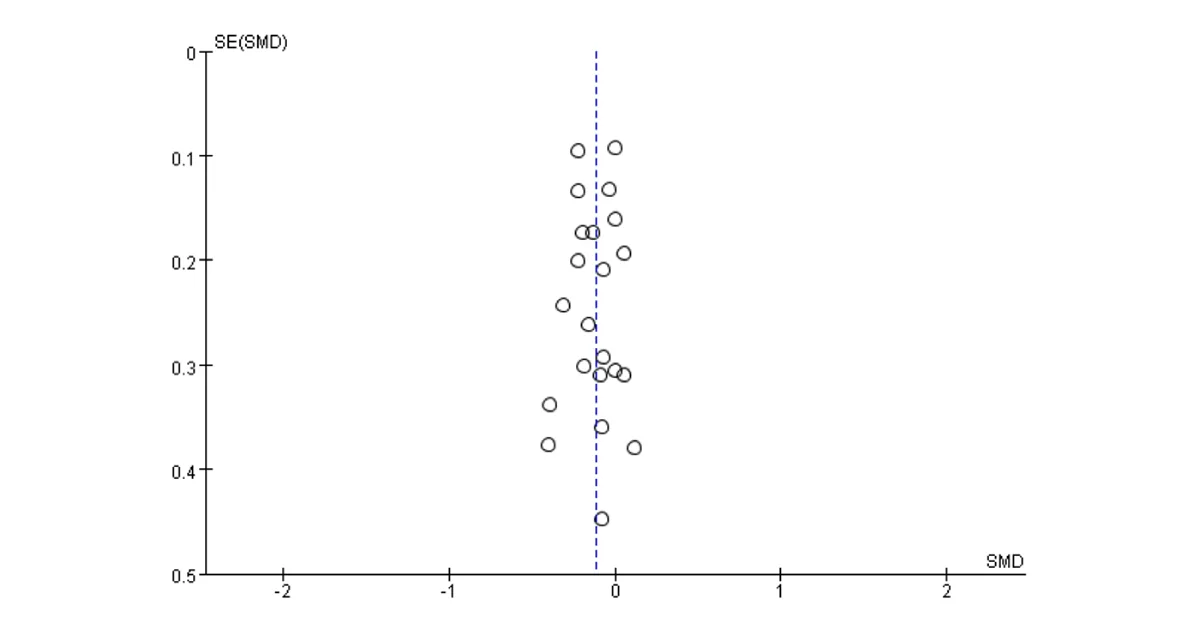
Funnel plot of standardized mean difference (SMD; Hedges g) against its SE (standard error). SMD: standardized mean difference.

#### Certainty of Evidence

The overall certainty of evidence for the primary outcome was further evaluated using the GRADE approach and was rated as moderate, with a 1-level downgrade for indirectness. Detailed GRADE ratings and justifications are provided in Multimedia Appendix 7.

### Synthesis of Secondary Outcomes

According to the predefined protocol, secondary outcomes included dietary behaviors, physical activity, and sedentary behavior. Quantitative synthesis was not conducted because these outcomes were assessed using diverse constructs, indicators, and measurement methods, limiting comparability across studies. In addition, only a small number of studies reported comparable quantitative data. Details of the secondary outcome measures, assessment tools, and results are presented in Multimedia Appendix 8.

#### Dietary Behaviors

Sixteen trials reported dietary behaviors. Five reported significantly greater improvements in at least one dietary outcome in the intervention group compared with controls, including higher overall diet quality and increased intake of core healthy foods [67], reduced total energy intake [43], improvements in healthy eating behaviors [58], decreases in high-fat meats and snack foods [53], and increased fruit and low-fat dairy intake with reduced sweets and processed meats [45]. The remaining 11 trials reported no differences in dietary behaviors between groups [3,41,48-52,55,59,61,68].

#### Physical Activity

Seventeen trials assessed physical activity outcomes. Two trials found significant between-group improvements favoring the intervention group, including greater increases in moderate-to-vigorous and vigorous physical activity with active video gaming [63], and longer daily and weekly physical activity duration with interventions delivered via websites [67]. The remaining 15 trials found no statistically significant between-group differences in physical activity outcomes [3,41,43,45,48-53,58,59,62,64,68].

#### Sedentary Behavior

Twelve trials evaluated outcomes related to sedentary behavior. Two trials reported significantly greater reductions in sedentary behavior in the intervention group compared with controls, including decreased average total sedentary time (minutes per day and per week) [67] and reduced hours of television or video game use at 6 months [3]. The remaining 10 trials found no differences in sedentary behavior outcomes between groups [41,45,49,50,52,53,55,56,59,68].

## Discussion

### Principal Findings

This systematic review and meta-analysis extends previous reviews by synthesizing evidence on the effects of parent-involved DHIs on adiposity-related outcomes and behaviors in children and adolescents with overweight or obesity while explicitly differentiating digital delivery modalities and parent-involved strategies. The meta-analytical results indicated a small improvement in BMI-related outcomes following parent-involved DHIs compared with control conditions (SMD=*−*0.11). Exploratory subgroup analyses did not identify statistically significant differences across digital delivery modalities or parent-involved strategies. In addition, descriptive synthesis found limited evidence of improvements in dietary behavior, physical activity, and sedentary behavior.

At the overall effect level, the small effect size observed in this review aligns with the broader evidence base in digital health research. A recent umbrella review reported a small effect (Cohen *d*=*−*0.28) of DHIs on BMI-related outcomes in children and adolescents [16]. It is important to note that these umbrella estimates were derived from a broad mix of digital and multicomponent interventions, some of which also incorporated parental or other supportive elements, resulting in substantial heterogeneity in intervention intensity and design. Consequently, the pooled effect is not directly comparable with the more narrowly defined parent-involved DHIs examined in the present review. Narrative reviews further suggest that benefits are more likely when digital delivery is combined with personalization, interactivity, and family involvement [69]. The pooled effect was numerically larger in trials using inactive controls (SMD=*−*0.15) than in those using active controls (SMD=*−*0.07), but the between-subgroup difference was not statistically significant and should not be interpreted as evidence of a difference in intervention effects by control type. Methodological guidance indicates that comparisons against active controls estimate relative effectiveness, whereas inactive controls can show whether an entire treatment package is effective in absolute terms [39]. Taken together, these findings underscore the importance of specifying and accounting for control group intensity when synthesizing intervention effects and interpreting the clinical significance of parent-involved DHIs for BMI-related outcomes.

The present review explored whether digital delivery modalities (eg, mobile phone apps or text messaging) moderated treatment effects. Although a statistically significant pooled effect was observed within the text messaging subgroup (k=2; SMD=−0.22), this exploratory finding does not establish that text messaging is more effective than other digital modalities. This observation aligns with prior evidence highlighting the potential of text messaging interventions in adolescent weight management [70]. Text messaging approaches are characterized by low cost, wide accessibility, and ease of implementation [71], but whether these characteristics translate into greater intervention effects remains uncertain.

The present review also explored whether parent-involved strategies moderated the effects of parent-involved DHIs. No statistically significant between-subgroup differences were observed for locus, action, or actor. Therefore, these findings should be interpreted cautiously as exploratory and do not establish the comparative superiority of any parent-involved strategy. Regarding locus, this dimension refers to the setting in which parental participation occurred rather than the physical location of digital intervention delivery, as digital technologies may enable intervention components to extend across multiple settings and transcend traditional physical boundaries. Previous reviews have also highlighted that interventions involving parental participation are effective in improving adiposity-related outcomes in children and adolescents across multiple settings, including school, home, and community environments [72,73].

When categorized by action, a statistically significant pooled effect was observed within the synchronous or quasi-synchronous subgroup, suggesting that external professional support may represent a potentially relevant feature warranting further investigation. The supportive accountability model proposes that real-time human support can enhance intervention adherence by establishing accountability, trust, and personalized assistance—functions difficult to achieve in fully automated programs [74]. However, real-time human support is often associated with higher labor and financial costs, which may limit the scalability and long-term sustainability of interventions [75]. Hybrid models that combine automated content with periodic human support therefore warrant further evaluation as a potential approach to balancing personalization and scalability.

By parental involvement actor, a statistically significant pooled effect was observed within the parent-focused subgroup, which is consistent with previous reviews highlighting the important role of parents in pediatric obesity interventions [72]. Because parents shape children’s and adolescents’ daily routines, dietary environments, and household rules, strengthening parental knowledge and skills may plausibly facilitate healthier family-level behaviors [76]. However, whether assigning parents a more central intervention role leads to greater effects remains uncertain and warrants direct comparison in adequately powered studies.

With respect to adiposity-related behaviors, parent-involved DHIs showed limited evidence for improvements in dietary behavior and physical activity or reductions in sedentary behavior. Significant between-group improvements were reported in 5 of 16 trials assessing dietary behaviors [43,45,53,58,67], compared with 2 of 17 trials for physical activity [63,67], and 2 of 12 trials for sedentary behavior [3,67]. This distribution may be related to the focus of the included interventions, as many parent-involved DHIs primarily targeted dietary modification, with comparatively fewer components designed to directly increase physical activity or reduce sedentary time. Similar trends have been reported in a systematic review, which reported improvements in dietary behaviors in 6 of 15 studies, compared with 1 of 11 for physical activity and 0 of 6 for sedentary behavior [17].

It is important to note that BMI-related outcomes and adiposity-related behaviors were synthesized using different approaches: the BMI-related finding was based on pooled effect sizes from 22 RCTs, whereas behavioral findings were synthesized descriptively according to whether individual studies reported significant between-group differences. Therefore, the apparent discrepancy between the small improvement in BMI-related outcomes and the limited behavioral findings should not be interpreted as evidence that BMI improved in the absence of behavioral change at the individual level. Adiposity-related behaviors were derived from different subsets of studies and assessed using heterogeneous measures and time points, with additional limitations related to self-report bias and cross-study comparability [77]. However, these findings cannot establish whether changes in the 3 behaviors examined fully account for the observed BMI-related effects. Family-level mechanisms, including parental weight-management practices, parent-child interactions, and the home environment, may also contribute [50,62,78]. This may partly explain why significant dietary improvements were reported in a larger proportion of trials, as eating behaviors are more directly shaped by parental practices and the home environment, whereas physical activity and sedentary behavior are also influenced by school schedules, peer relationships, and community environments [79]. Future studies should therefore examine parental and family-level mechanisms alongside the lifestyle behaviors of children and adolescents to better clarify how parent-involved DHIs influence adiposity-related outcomes.

### Limitations

Several limitations should be considered. First, although statistically significant, the pooled effect on BMI-related outcomes among children and adolescents with overweight or obesity was small, which should be considered when interpreting its clinical relevance. Second, subgroup analyses were exploratory and limited by a small number of studies in some categories (eg, only 2 trials for text messaging), and the related findings should be interpreted cautiously. Third, although intervention duration was reported across all studies, the largely unreported session frequency and length precluded total intervention dose and dose-response analysis. Meanwhile, variation in follow-up schedules limited assessment of whether the observed effects were sustained over time. Fourth, secondary outcomes could not be quantitatively synthesized because of substantial heterogeneity in outcome measures and assessment methods. Finally, individual studies often included broad age ranges, limiting our ability to reliably examine age-specific effects. Given that parental influence, autonomy, and patterns of technology use may differ across developmental stages, the pooled effect may not apply uniformly from childhood through adolescence.

### Implications

The findings of this review have several implications for both practice and future research. Parent-involved DHIs might represent a feasible and scalable adjunct to existing pediatric obesity management strategies. However, because no statistically significant differences were detected across digital delivery modalities or parent-involved strategies, the within-subgroup patterns observed for text messaging, parent-focused approaches, and synchronous or quasi-synchronous support should be regarded as hypothesis-generating rather than as evidence for prioritizing specific intervention strategies. Future trials should directly compare these characteristics and evaluate their feasibility, engagement, and effectiveness. For future research, improvements in methodological rigor are warranted. Clearer reporting of allocation concealment, blinding procedures, and adherence to intervention protocols would reduce potential bias and enhance internal validity. In addition, given the variability observed across control types and intervention formats, future trials should explicitly justify comparator selection and report intervention intensity to allow more precise interpretation of relative effectiveness.

### Conclusions

This systematic review and meta-analysis indicate that parent-involved DHIs may produce a small but significant improvement in BMI-related outcomes among children and adolescents with overweight or obesity, supported by moderate-certainty evidence. However, evidence for improvements in adiposity-related behaviors remains limited, and neither the optimal digital delivery modality nor the most effective parent-involved strategy has been established. More rigorously designed RCTs with clearly defined control types, standardized reporting, and longer follow-up are needed to clarify the durability of effects and identify optimal combinations of delivery modalities and parent-involved strategies.

## Appendix

Multimedia Appendix 1 PRISMA checklist.

Multimedia Appendix 2 Search strategy.

Multimedia Appendix 3 Operational definitions and classification criteria.

Multimedia Appendix 4 Reasons for full-text exclusion and inclusion.

Multimedia Appendix 5 Risk of bias assessment (RoB 2).

Multimedia Appendix 6 Sensitivity analysis.

Multimedia Appendix 7 GRADE ratings and reasons.

Multimedia Appendix 8 Secondary outcome characteristics.

## Abbreviations

BMI%p95: BMI as a percent of the 95th percentile
DHI: digital health intervention
GRADE: Grading of Recommendations, Assessment, Development, and Evaluation
NICE: National Institute for Health and Care Excellence
PICOS: Population, Intervention, Comparison, Outcomes, and Study Design
PRISMA: Preferred Reporting Items for Systematic Reviews and Meta-Analyses
PROSPERO: International Prospective Register of Systematic Reviews
RCT: randomized controlled trial
RoB 2: Cochrane Risk of Bias tool for randomized trials
SMD: standardized mean difference
WC: waist circumference
WHR: waist height ratio

## Data Availability

All data generated or analyzed during this study are included in this paper and its supplementary information files.
