# Supplementary material for: Effects of Parent-Involved Digital Health Interventions on Adiposity-Related Outcomes and Behaviors in Children and Adolescents With Overweight or Obesity: Systematic Review and Meta-Analysis
Source: J Med Internet Res. 2026 Oct 30;28:e95757. doi: 10.2196/95757 (PMC13768816; doi:10.2196/95757)
Supplement: Supplementary file 2 [file jmir_v28i1e95757_app3.docx]

**Table S1.** Operational definitions of DHI delivery modalities.

| Digital delivery modality | Operational definition | Classification criteria in this review |
| --- | --- | --- |
| Mobile phone applications | Digital interventions delivered through software applications used on smartphones or tablets to provide intervention content, self-monitoring, feedback, behavioral guidance, or other intervention functions. | Classified as mobile phone applications when a mobile application was identified in the original report as the primary digital platform through which participants accessed or interacted with the intervention. |
| Text messaging | Digital interventions in which text-based messages, including SMS or equivalent mobile messaging, are used to deliver intervention information, reminders, prompts, feedback, or behavioral support. | Classified as text messaging when text-based communication constituted the primary digital channel for delivering intervention content or support, regardless of whether the messages were received on a mobile phone also capable of running applications. |
| Websites | Digital interventions delivered through web-based platforms that provide access to intervention information, educational materials, monitoring, feedback, or interactive intervention activities. | Classified as websites when a web-based platform was the principal digital interface through which participants accessed intervention content or activities. |
| Telehealth | Remote delivery of professional health care, counseling, monitoring, or guidance through information and communication technologies, which may include telephone, video, web-based, or other remote communication channels. | Classified as telehealth when the original study characterized the core digital component as telehealth, telemedicine, or remote professional care, and the principal function of the technology was to enable remote interaction, counseling, clinical support, or monitoring between participants and health professionals. Because telehealth may be delivered through different communication channels, classification was based on the intervention model and function described by the study authors rather than on a specific device alone. |
| Telephone | Digital or technology-assisted interventions in which voice-based telephone calls or interactive voice response are used to deliver counseling, coaching, intervention content, or behavioral support. | Classified as telephone when telephone calls or interactive voice response constituted the primary digital delivery channel and the intervention was described primarily as telephone-based coaching, counseling, or automated voice support rather than as a broader telehealth or telemedicine model. |
| Exergaming / active video gaming | Digital interventions that combine video gaming with physical activity, typically requiring body movement to interact with the gaming system and thereby promote physical activity engagement. | Classified as exergaming/active video gaming when movement-based interactive gaming constituted the principal digital component used to promote physical activity or support weight-management behaviors. |
| Hybrid combination interventions | Interventions integrating two or more distinct digital delivery modalities as substantive components of the intervention. | Classified as hybrid combination interventions when two or more digital delivery modalities contributed substantively to intervention delivery and no single modality adequately represented the overall digital delivery approach. |

Note. Digital delivery modalities were operationally classified according to the primary digital channel and intervention function described in the original studies. Because some modalities may overlap conceptually—for example, telehealth may be delivered through telephone, video, or web-based communication—categories in this review were not defined solely by the hardware used. Interventions were classified according to the modality that best represented their primary delivery model; when two or more modalities contributed substantively and no single modality adequately represented the intervention, the study was classified as a hybrid combination intervention.

**Table S2.** Operational definitions of parent-involved strategies.

| Digital delivery modality | | Operational definition | Classification criteria in this review |
| --- | --- | --- | --- |
| Locus | Home-based | Parental involvement is primarily conducted within the home environment | Classified as home-based when parent participation occurred exclusively within the home setting. |
|  | Outpatient and home-based | Parental involvement occurs through a combination of professional contact in outpatient settings and intervention activities conducted at home. | Classified as outpatient and home-based when interventions included both healthcare/clinical contacts and home-based parental participation. |
|  | School/community and home-based | Parental involvement involves both community or school-based intervention activities and home-based participation. | Classified as school/community and home-based when interventions combined school/community delivery with parental involvement at home. |
| Style | Formal | Parental participation is intentionally incorporated into a structured intervention program, with predefined responsibilities, activities, guidance, or procedures. | All included interventions were classified as formal involvement because structured parental participation was required as an eligibility criterion. This dimension was described but not examined as an effect modifier. |
| Action | Self-directed completion | Parents independently access intervention materials or complete assigned activities without direct interaction with intervention personnel. | Classified as self-directed completion when parental participation mainly involved independent implementation of intervention tasks, monitoring, or educational activities. |
|  | Synchronous/quasi-synchronous interaction | Parents receive direct interaction, communication, feedback, or guidance from intervention personnel in real time or near real time. | Classified as synchronous/quasi-synchronous interaction when scheduled communication, counseling, coaching, or reciprocal interaction between parents and intervention providers was included. |
|  | Fully indirect/non-interactive | Parents participate indirectly through intervention structures or child-focused components without independently completing intervention tasks or interacting with intervention personnel. | Classified as fully indirect/non-interactive when parents provided support through the intervention framework but did not receive direct intervention components or communication. |
| Actor | Parent-focused | Parents are the primary targets of intervention content, with intervention components mainly designed to modify parental knowledge, skills, or behaviors related to child weight management. | Classified as parent-focused when parents were the primary recipients of intervention content and children were not the main intervention targets. |
|  | Parent-led joint | Parents and children participate jointly, with parents acting as active agents who support or implement intervention strategies with their children. | Classified as parent-led joint when both parents and children were involved and parents had a central role in facilitating behavior change. |
|  | Child- and adolescent-targeted and parent support | Children or adolescents are the primary intervention targets, while parents provide supportive roles during intervention implementation. | Classified as child- and adolescent-targeted and parent support when intervention content was mainly directed toward children/adolescents but parental involvement was included as supportive assistance. |

Note. Parent-involved strategies were classified according to the multidimensional framework of parental involvement proposed by Driessen (2021), including locus, style, action, and actor. Within each dimension, categories were further specified based on the characteristics of parent-involved digital health interventions targeting childhood and adolescent weight management.

**References**

6. Digital Implementation Investment Guide (DIIG): Integrating Digital Interventions into Health Programmes. World Health Organization. Geneva. World Health Organization; 2020. URL: https://iris.who.int/handle/10665/334306 [accessed 2026-10-22]

16. Flølo TN, Tørris C, Riiser K, Almendingen K, Chew HSJ, Fosså A, et al. Digital health interventions to treat overweight and obesity in children and adolescents: an umbrella review. Obes Rev. 2025;26(6):e13905. [doi: 10.1111/obr.13905] [Medline: 39972996]

24. Schoeppe S, Alley S, Van Lippevelde W, Bray NA, Williams SL, Duncan MJ, et al. Efficacy of interventions that use apps to improve diet, physical activity and sedentary behaviour: a systematic review. Int J Behav Nutr Phys Act. Dec 07, 2016;13(1):127. [FREE Full text] [doi: 10.1186/s12966-016-0454-y] [Medline: 27927218]

25. Cole-Lewis H, Kershaw T. Text messaging as a tool for behavior change in disease prevention and management. Epidemiol Rev. 2010;32(1):56-69. [FREE Full text] [doi: 10.1093/epirev/mxq004] [Medline: 20354039]

26. Barak A, Klein B, Proudfoot JG. Defining internet-supported therapeutic interventions. Ann Behav Med. Aug 2009;38(1):4-17. [doi: 10.1007/s12160-009-9130-7] [Medline: 19787305]

27. Goode AD, Reeves MM, Eakin EG. Telephone-delivered interventions for physical activity and dietary behavior change: an updated systematic review. Am J Prev Med. Jan 2012;42(1):81-88. [doi: 10.1016/j.amepre.2011.08.025] [Medline: 22176852]

28. LeBlanc AG, Chaput J-P, McFarlane A, Colley RC, Thivel D, Biddle SJH, et al. Active video games and health indicators in children and youth: a systematic review. PLoS One. 2013;8(6):e65351. [FREE Full text] [doi: 10.1371/journal.pone.0065351] [Medline: 23799008]

14. Driessen G. Parental involvement: types and effects. Ann Sofia Univ "St Kliment Ohridski" Fac Educ Stud Arts Book of Educ Stud. 2021;114(1):7-29. [FREE Full text]

29. Golan M, Crow S. Parents are key players in the prevention and treatment of weight-related problems. Nutr Rev. Jan 2004;62(1):39-50. [doi: 10.1111/j.1753-4887.2004.tb00005.x] [Medline: 14995056]

30. Golan M. Parents as agents of change in childhood obesity—from research to practice. Int J Pediatr Obes. 2006;1(2):66-76. [doi: 10.1080/17477160600644272] [Medline: 17907317]

15. Hammersley ML, Jones RA, Okely AD. Parent-focused childhood and adolescent overweight and obesity eHealth interventions: a systematic review and meta-analysis. J Med Internet Res. 2016;18(7):e203. [FREE Full text] [doi: 10.2196/jmir.5893] [Medline: 27443862]

31. Davison KK, Lawson HA, Coatsworth JD. The Family-centered Action Model of Intervention Layout and Implementation (FAMILI): the example of childhood obesity. Health Promot Pract. Jul 2012;13(4):454-461. [doi: 10.1177/1524839910377966] [Medline: 21632465]

32. Yardley L, Morrison L, Bradbury K, Muller I. The person-based approach to intervention development: application to digital health-related behavior change interventions. J Med Internet Res. Jan 30, 2015;17(1):e30. [FREE Full text] [doi: 10.2196/jmir.4055] [Medline: 25639757]
