# Supplementary material for: Effects of Parent-Involved Digital Health Interventions on Adiposity-Related Outcomes and Behaviors in Children and Adolescents With Overweight or Obesity: Systematic Review and Meta-Analysis
Source: J Med Internet Res. 2026 Oct 30;28:e95757. doi: 10.2196/95757 (PMC13768816; doi:10.2196/95757)
Supplement: Supplementary file 3 [file jmir_v28i1e95757_app6.docx]

**Figure S1.** Sensitivity analysis restricted to trials reporting BMI z-score [3,43-53,55,56,58,59,61,63,64,67].

**Figure S2.** Sensitivity analysis restricted to trials published from 2016 to 2025 [3,43-47,49-51,56,58,59,61,62,64,67].

**References**

3.	Pbert L, Druker S, Crawford S, Frisard C, Bram J, Olendzki B, et al. A pediatric primary care practice-based obesity intervention to support families: a cluster-randomized clinical trial. Obesity (Silver Spring). 2024;32(9):1721-1733. [doi: 10.1002/oby.24100] [Medline: 39081043]

43.	Chai LK, Collins CE, May C, Ashman A, Holder C, Brown LJ, et al. Feasibility and efficacy of a web-based family telehealth nutrition intervention to improve child weight status and dietary intake: a pilot randomised controlled trial. J Telemed Telecare. 2021;27(3):146-158. [doi: 10.1177/1357633X19865855] [Medline: 31364474]

44.	Jones AR, Mann KD, Cutler LR, Pearce MS, Tovée MJ, Ells LJ, et al. A randomised control trial investigating the efficacy of the MapMe intervention on parental ability to correctly categorise overweight in their child and the impact on child BMI Z-score change at 1 year. Children (Basel). 2023;10(9):1577. [FREE Full text] [doi: 10.3390/children10091577] [Medline: 37761538]

45.	Varagiannis P, Magriplis E, Risvas G, Vamvouka K, Nisianaki A, Papageorgiou A, et al. Effects of three different family-based interventions in overweight and obese children: the "4 your family" randomized controlled trial. Nutrients. 2021;13(2):341. [FREE Full text] [doi: 10.3390/nu13020341] [Medline: 33498894]

46.	Ahmad N, Shariff ZM, Mukhtar F, Lye M. Family-based intervention using face-to-face sessions and social media to improve Malay primary school children's adiposity: a randomized controlled field trial of the Malaysian REDUCE programme. Nutr J. 2018;17(1):74. [FREE Full text] [doi: 10.1186/s12937-018-0379-1] [Medline: 30071855]

47.	Baños RM, Oliver E, Navarro J, Vara MD, Cebolla A, Lurbe E, et al. Efficacy of a cognitive and behavioral treatment for childhood obesity supported by the ETIOBE web platform. Psychol Health Med. 2019;24(6):703-713. [doi: 10.1080/13548506.2019.1566622] [Medline: 30648879]

48.	Davis AM, Sampilo M, Gallagher KS, Landrum Y, Malone B. Treating rural pediatric obesity through telemedicine: outcomes from a small randomized controlled trial. J Pediatr Psychol. 2013;38(9):932-943. [FREE Full text] [doi: 10.1093/jpepsy/jst005] [Medline: 23428652]

49.	Davis A, Lancaster B, Fleming K, Romine RS, Forseth B, Nelson E, et al. Effectiveness of a paediatric weight management intervention for rural youth (iAmHealthy): primary outcomes of a cluster randomised control trial. Pediatr Obes. 2024;19(3):e13094. [doi: 10.1111/ijpo.13094] [Medline: 38173133]

50.	Estabrooks PA, Shoup JA, Gattshall M, Dandamudi P, Shetterly S, Xu S. Automated telephone counseling for parents of overweight children: a randomized controlled trial. Am J Prev Med. 2009;36(1):35-42. [doi: 10.1016/j.amepre.2008.09.024] [Medline: 19095163]

51.	Fleischman A, Hourigan SE, Lyon HN, Landry MG, Reynolds J, Steltz SK, et al. Creating an integrated care model for childhood obesity: a randomized pilot study utilizing telehealth in a community primary care setting. Clin Obes. 2016;6(6):380-388. [FREE Full text] [doi: 10.1111/cob.12166] [Medline: 27863024]

52.	Maddison R, Marsh S, Foley L, Epstein LH, Olds T, Dewes O, et al. Screen-Time Weight-Loss Intervention Targeting Children at Home (SWITCH): a randomized controlled trial. Int J Behav Nutr Phys Act. 2014;11:111. [FREE Full text] [doi: 10.1186/s12966-014-0111-2] [Medline: 25204320]

53.	Nguyen B, Shrewsbury VA, O'Connor J, Steinbeck KS, Lee A, Hill AJ, et al. Twelve-month outcomes of the Loozit randomized controlled trial: a community-based healthy lifestyle program for overweight and obese adolescents. Arch Pediatr Adolesc Med. 2012;166(2):170-177. [doi: 10.1001/archpediatrics.2011.841] [Medline: 22312175]

55.	Wright JA, Phillips B, Watson B, Newby P, Norman G, Adams W. Randomized trial of a family-based, automated, conversational obesity treatment program for underserved populations. Obesity (Silver Spring). 2013;21(9):E369-E778. [doi: 10.1002/oby.20388] [Medline: 23512915]

56.	Wald ER, Ewing LJ, Moyer SCL, Eickhoff JC. An interactive web-based intervention to achieve healthy weight in young children. Clin Pediatr (Phila). 2018;57(5):547-557. [FREE Full text] [doi: 10.1177/0009922817733703] [Medline: 29067819]

58.	Umano GR, Masino M, Cirillo G, Rondinelli G, Massa F, di Santo Stefano GSRCM, et al. Effectiveness of smartphone app for the treatment of pediatric obesity: a randomized controlled trial. Children (Basel). 2024;11(10):1178. [FREE Full text] [doi: 10.3390/children11101178] [Medline: 39457143]

59.	Tugault-Lafleur CN, De-Jongh González O, Macdonald J, Bradbury J, Warshawski T, Ball GDC, et al. Efficacy of the Aim2Be intervention in changing lifestyle behaviors among adolescents with overweight and obesity: randomized controlled trial. J Med Internet Res. 2023;25:e38545. [FREE Full text] [doi: 10.2196/38545] [Medline: 37097726]

61.	Fiechtner L, Kleinman K, Melly SJ, Sharifi M, Marshall R, Block J, et al. Effects of proximity to supermarkets on a randomized trial studying interventions for obesity. Am J Public Health. 2016;106(3):557-562. [doi: 10.2105/AJPH.2015.302986] [Medline: 26794159]

63.	Trost SG, Sundal D, Foster GD, Lent MR, Vojta D. Effects of a pediatric weight management program with and without active video games a randomized trial. JAMA Pediatr. 2014;168(5):407-413. [doi: 10.1001/jamapediatrics.2013.3436] [Medline: 24589566]

64.	Damayanti D, Ngadiarti I, Mega MDP, Jaceldo-Siegl K. Online intervention of elementary school children with overweight and obese during pandemic in Jakarta: a pilot cluster randomized control trial. Nutr Clín Diet Hosp. 2025;45(2):251-259. [doi: 10.12873/766damayanti]

67.	Zhu D, Dordevic AL, Gibson S, Davidson ZE. The effectiveness of a 10-week family-focused e-Health healthy lifestyle program for school-aged children with overweight or obesity: a randomised control trial. BMC Public Health. 2025;25(1):59. [FREE Full text] [doi: 10.1186/s12889-024-21120-5] [Medline: 39773158]

62.	Kim HS, Park J, Park K, Lee M, Ham OK. Parent involvement intervention in developing weight management skills for both parents and overweight/obese children. Asian Nurs Res (Korean Soc Nurs Sci). 2016;10(1):11-17. [FREE Full text] [doi: 10.1016/j.anr.2015.07.006] [Medline: 27021829]
