# Supplementary material for: Effects of Parent-Involved Digital Health Interventions on Adiposity-Related Outcomes and Behaviors in Children and Adolescents With Overweight or Obesity: Systematic Review and Meta-Analysis
Source: J Med Internet Res. 2026 Oct 30;28:e95757. doi: 10.2196/95757 (PMC13768816; doi:10.2196/95757)
Supplement: Supplementary file 4 [file jmir_v28i1e95757_app7.docx]

Detailed GRADE ratings and reasons

| **Certainty assessment** | | | | | | | **№ of patients** | | **Effect** | | **Certainty** | **Importance** |
| --- | --- | --- | --- | --- | --- | --- | --- | --- | --- | --- | --- | --- |
| **№ of studies** | **Study design** | **Risk of bias** | **Inconsistency** | **Indirectness** | **Imprecision** | **Other considerations** | **BMI Z** | **placebo** | **Relative (95% CI)** | **Absolute (95% CI)** |  |  |
| **BMI** | | | | | | | | | | | | |
| 22 | randomized trials | not serious | serious | serious^a^ | not serious | none | 1379 | 1254 | - | SMD **0.11 SD lower** (0.19 lower to 0.04 lower) | ⨁⨁⨁◯ Moderate^a^ | CRITICAL |

Note. CI, confidence interval; SMD, standardized mean difference

^a^ Downgraded for indirectness as most interventions were multicomponent and heterogeneous.
